# Are physicians ready for precision antibiotic prescribing? A qualitative analysis of the acceptance of artificial intelligence-enabled clinical decision support systems in India and Singapore

**DOI:** 10.1016/j.jgar.2023.08.016

**Published:** 2023-12

**Authors:** Zhilian Huang, Mithun Mohan George, Yi-Roe Tan, Karthiga Natarajan, Emily Devasagayam, Evonne Tay, Abi Manesh, George M. Varghese, Ooriapadickal Cherian Abraham, Anand Zachariah, Peiling Yap, Dorothy Lall, Angela Chow

**Affiliations:** a Infectious Diseases Research and Training Office, National Centre for Infectious Diseases, Singapore; b Department of Preventive and Population Medicine, Office of Clinical Epidemiology, Analytics, and Knowledge [OCEAN], Tan Tock Seng Hospital, Singapore; c Department of Infectious Diseases, Christian Medical College, Vellore, Tamil Nadu, India; d International Digital Health & AI Research Collaborative (I-DAIR), Geneva, Switzerland; e Department of Medicine, Christian Medical College, Vellore, Tamil Nadu, India; f Department of Community Health, Christian Medical College Vellore - Chittoor Campus, Andhra Pradesh, India; g Lee Kong Chian School of Medicine, Nanyang Technological University, Singapore; h Saw Swee Hock School of Public Health, National University of Singapore, Singapore

**Keywords:** Antimicrobial resistance, Artificial intelligence, Clinical decision support system, Antibiotic prescribing

## Abstract

**Objectives:**

Artificial intelligence (AI)-driven clinical decision support systems (CDSSs) can augment antibiotic decision-making capabilities, but physicians’ hesitancy in adopting them may undermine their utility. We conducted a cross-country comparison of physician perceptions on the barriers and facilitators in accepting an AI-enabled CDSS for antibiotic prescribing.

**Methods:**

We conducted in-depth interviews with physicians from the National Centre for Infectious Diseases (NCID), Singapore, and Christian Medical College Vellore (CMCV), India, between April and December 2022. Our semi-structured in-depth interview guides were anchored on Venkatesh's UTAUT model. We used clinical vignettes to illustrate the application of AI in clinical decision support for antibiotic prescribing and explore medico-legal concerns.

**Results:**

Most NCID physicians felt that an AI-enabled CDSS could facilitate antibiotic prescribing, while most CMCV physicians were sceptical about the tool's utility. The hesitancy in adopting an AI-enabled CDSS stems from concerns about the lack of validated and successful examples, fear of losing autonomy and clinical skills, difficulty of use, and impediment in work efficiency. Physicians from both sites felt that a user-friendly interface, integration with workflow, transparency of output, a guiding medico-legal framework, and training and technical support would improve the uptake of an AI-enabled CDSS.

**Conclusion:**

In conclusion, the acceptance of AI-enabled CDSSs depends on the physician's confidence with the tool's recommendations, perceived ease of use, familiarity with AI, the organisation's digital culture and support, and the presence of medico-legal governance of AI. Progressive implementation and continuous feedback are essential to allay scepticism around the utility of AI-enabled CDSSs.

## Highlights

• Singapore physicians were mostly accepting of AI tools for antibiotic prescribing.
• India physicians were skeptical about the utility and value of AI tools.
• Practice context and digital culture shape physicians’ acceptance of AI tools.
• Validating AI tools will instill physicians’ confidence in adopting them.
• Medico-legal oversight of AI may dispel physicians’ hesitancy in adopting AI tools.

## 1 Introduction

Antimicrobial resistance (AMR) poses a serious threat to public health and clinical care [1]. Antimicrobial resistance occurs when microorganisms become resistant to drugs intended to inhibit them, rendering human infections more severe and deadly [2]. In 2019, 1.27 million global deaths were directly attributed to bacterial AMR [3], with misuse and overuse of antibiotics being the major drivers of AMR [4,5]. Deaths attributable to AMR were disproportionately higher in low- and middle-income countries (LMICs) because of the higher burden of communicable diseases [6]. For example, India has one of the highest burdens of AMR in the world due to injudicious use of antimicrobials and environmental contamination [7].

Physicians make their prescribing decisions by first making a presumptive diagnosis, identifying the right drug for the suspected pathogen, regulating the dose and frequency of the antimicrobial, and determining the best administration route to achieve the desired drug level at the site of infection. Although the theory is simple, it is difficult and complex to execute in practice because of the need to consider various clinical and non-clinical factors. Factors such as unclear discriminatory clinical signs and symptoms, limited differentiating biomarkers, and the lack of cheap and accessible point-of-care microbiologic diagnostic tests lead to diagnostic uncertainty [8]. Hence, there is a tendency for physicians to over-prescribe antibiotics because of decision fatigue, uncertainty avoidance, and to meet patient expectations [9], [10], [11].

Hospital antimicrobial stewardship programs were found to improve antibiotic use and antibiotic prescribing without compromising patient safety [12]. Rules-based clinical decision support systems (CDSSs) were developed to enhance clinical decisions, improve clinical outcomes, and reduce mortality by guiding doctors in selecting the most appropriate antibiotics for empiric therapy [13], [14], [15], [16]. However, many physicians still prefer to exercise their professional judgement and tend to override the recommendations of CDSSs in complex patient situations [17,18].

More recently, artificial intelligence (AI)-driven technologies have demonstrated superiority in capturing and analysing complex health problems and are increasingly used in medicine to augment decision-making [19]. As such, AI-enabled CDSSs leveraging machine learning present excellent opportunities for a complex matrix of data, such as clinical severity, comorbidities, host immune status, prior antibiotic use, local resistance profile, and more, to be analysed prior to the prescription of appropriate antibiotics. However, many factors determine the feasibility and acceptability of such systems, such as the lack of trust in AI-derived recommendations [20], [21], [22]. Therefore, it is imperative to understand physicians' knowledge, attitudes, perceptions, and acceptance of AI-enabled CDSSs to guide the future design and adoption of such tools. Given the paucity of cross-country studies [23], which are important for the scalability of such tools in the future, we aimed to explore the perspectives of physicians in India and Singapore to understand the barriers and facilitators to the acceptance of AI-enabled CDSSs for antibiotic prescribing in different socio-cultural and practice contexts.

## 2 Methods and materials

### 2.1 Study context

#### 2.1.1 India

Christian Medical College Vellore (CMCV) is a private hospital, medical centre, and research institute located in Tamil Nadu. The 3500-bed institution offers medical services across multiple specialities from primary to tertiary care. The medical centre, equipped with state-of-the-art technologies for diagnosis and treatment, is India's largest referral centre for hematologic diseases and bone marrow transplants. Antimicrobial susceptibility patterns are closely monitored by the institution and regularly updated through guideline publications. There is no CDSS in the institution currently (Supplemental Text S1A).

#### 2.1.2 Singapore

The National Centre for Infectious Diseases (NCID), co-located with Tan Tock Seng Hospital (TTSH), is a 330-bed purpose-built facility that serves as the designated national centre for management of emerging infectious diseases in Singapore. The facility is equipped with comprehensive diagnostic capabilities and state-of-the-art healthcare technology to enhance care delivery to patients. Physicians working in NCID use the same electronic medical record system as TTSH, which includes a rules-based antibiotic CDSS named ‘ARUS-C’ (Supplemental Text S1B).

### 2.2 Study design

We conducted in-depth interviews (IDIs) with physicians working in the inpatient setting between April and December 2022. Physicians were sampled from NCID, Singapore and CMCV, India. We purposively sampled physicians with varying seniority and pace of innovation adoption to ensure maximum variation. Our semi-structured IDI guides (Supplemental Table S4) were anchored on Venkatesh's Unified Theory of Acceptance and Use of Technology (UTAUT) model [24], piloted with a few physicians, and adapted to ensure relevance to each country's context.

### 2.3 In-depth interviews

The IDIs, comprising an interviewer and a note-taker, were conducted face-to-face or via video conferencing (i.e., Zoom). We progressively filled a matrix framework with interview notes to assess for data saturation. The NCID interviews included a vignette to demonstrate the differences between the rules-based CDSS familiar to physicians and a future-state AI-enabled CDSS with augmented diagnostic capabilities (Supplemental Vignette S2). The CMCV interviews included a vignette comprising case scenarios to describe the workings of an AI-enabled CDSS (Supplemental Vignette S3).

Next, we explored physicians’ perceptions of the performance and effort expectancies of using an AI-enabled CDSS, the effect of social influence on its adoption, and the conditions that facilitate its adoption. We also discussed the ethical considerations and medico-legal concerns on the adoption of an AI-enabled CDSS and asked physicians about features of the tool that would facilitate their uptake.

### 2.4 Analysis

Interviews were audio-recorded, transcribed verbatim by an external party, and checked by study team members. Study team members coded the transcripts using the deductive-inductive-deductive framework method [25]. We achieved data familiarization through notetaking during the interviews and transcript reviews. After which, all coders independently performed deductive coding on one transcript and calibrated the coding during team meetings. Coding was conducted by two and three independent researchers in Singapore and India, respectively. New categories that arose from the coding were discussed during team meetings, and disagreements were resolved with reasoning and team voting. We also conducted regular meetings between both sites to ensure agreement with the process and codes. After finalizing the coding categories, essential themes from both countries were charted into a thematic matrix and juxtaposed to highlight the similarities and differences between the two settings. NVivo 12 Pro was used to organize the qualitative data [26].

## 3 Results

We interviewed 30 and 15 physicians in CMCV and NCID, respectively. The baseline characteristics of the physicians are presented in Table 1. Table 2 shows the component definitions of the UTAUT model and the themes and representative quotes from the India and Singapore interviews. The major themes from our analysis were aligned with the UTAUT model. Physicians’ desired features for the AI tool are classified under facilitating conditions. We additionally included ethical considerations in Table 2.

**Table 1:** Demographic characteristics of participants in India and Singapore.

Demographic characteristics	No. of participants	
	India (N=30)	Singapore (N=15)
**Age (in y)**		
Median (Min, Max)	37.5 (31, 61)	32 (26, 64)
**Years of practice as a physician, n (%)**		
< 2	0	2 (13.3)
2–5	5 (16.7)	2 (13.3)
>5–9	7 (23.3)	3 (20.0)
> 9	18 (60.0)	8 (53.3)
**Sex, n (%)**		
Male	21 (70.0)	9 (60.0)
Female	9 (30.0)	6 (40.0)
**Basic medical education, n (%)**		
Local	30 (100.0)	9 (60.0)
Overseas	0	6 (40.0)
**Professional designation, n (%)**		
Medical Officers/ Residents/Senior Residents	0	8 (53.3)
Senior Resident Physicians	3 (10.0)	1 (6.7)
Consultants/Assistant and associate professors	18 (60.0)	3 (20.0)
Senior Consultants/Professors)	9 (30.0)	3 (20.0)
**Specialty of practice, n (%)**		
Nephrology and Urology	3 (10.0)	0
Infectious Diseases	5 (16.7)	15 (100)
Respiratory medicine	3 (10.0)	0
Critical care (Medical & Surgical)	7 (23.3)	0
Haematology	3 (10.0)	0
Other medical specialities*	9 (30.0)	0

*NOTE.* Other medical specialties include general medicine (3), gastroenterology and hepatology (2), rheumatology, endocrinology, dermatology, and orthopaedics.

**Table 2:** Themes and representative quotes from the India and Singapore interviews.

Themes	Singapore NCID representative Quotes	India CMC Vellore representative Quotes
**Performance expectancy** (The degree an individual believes that the system helps improve job performance)		

**Perceived usefulness of AI-enabled CDSS** **Singapore:** Physicians feel that an AI-enabled CDSS would help them to arrive at decisions faster and serves as a tool to support their antibiotic prescribing decision. **India:** Most physicians are sceptical of the AI-enabled CDSS as they felt that it would not add value to physicians' capabilities.	“Oh well if it's fast enough, it will definitely be a lot quicker, because now we have to consider all those different things by ourselves right without a system in place but if the system can do it for you and it already takes all that into consideration, it will be much easier.” (Medical Officer) “I would use it for the purposes of complicated cases, I think it's useful to always have a check against my biases.” (Medical Officer)	“I'm slightly sceptical about it. But I know people who've worked in that, and they say that it helps in that. Yeah, so in the papers that we read, they use a lot of AI data.” (Consultant) “Short answer, it sounds good. But at a practical level, I don't know to what extent that can be useful. I'll tell you the reason because, like for instance, even in our setup, one thing is the huge burden of comorbidities, which most of our patients will have.” (Consultant)
**AI-enabled CDSS may impair clinical judgement** Physicians expressed concern that the use of such tools may encourage over-reliance on the use of the system and diminish their ability to make antibiotic prescribing decisions.	“As a physician, there is a temptation to just rely on AI and basically just fall back on it.” (Medical Officer) “I'm just scared that the junior[s] will just use it without thinking, without reasoning, you know? [The tool] will stop people from learning and reasoning. (Senior Consultant).”	“I think over time people won't know how to decide. It will make people, doctors dumb, over a period of time, because everyone will just key in and what is the AI telling, ok I'll prescribe that. It's over time.” (Senior Consultants) “The thing is, I think that it's not that good for our trainees, because they are not able to make those decisions without the aid of that machine.” (Senior Consultant)
**AI-enabled CDSS may impede work efficiency** Junior physicians felt that an AI-enabled CDSS might slow down the entire workflow. The need to bypass the system becomes an impediment to their daily workflow if the recommendations are not deemed to be accurate.	“I may need to type in the information and wait for the system to run. You'll get a bit irritated, because now you need even more input, and most of such black box [systems] they need a bit more computational power so it might be even slower as opposed to a rule-based ARUS-C. So, my main worry is that it slows down the workflow even more.” (Medical Officer)	“That's all based on how efficiently you put it together. AI can slow things; AI can fasten things up.” (Senior Consultant)

**Effort expectancy** (The ease of use and complexity of the system)		

**Familiarisation with a new system** Physicians anticipate having to put in more effort to get used to the system at the beginning.	“Oh well, I've been changing computer systems every six months … It's definitely difficult, but you're forced to be functional within the first week. Then you get a bit better at the first month mark.” (Medical Officer)	“I don't like to begin, to work with any new system, initially maybe a difficult one, but anything if it's got integrated and if the interface is good, we, we will go for it.” (Senior Resident)
**An apprehension of the time required to use the system** **Singapore:** Two junior physicians mentioned that they would require more effort to input data into the AI system instead of concluding the case quickly if it is straightforward. **India:** Most physicians expressed that while they were familiar with the application of AI, they could not visualise how such a tool could be integrated into their everyday work.	“It seems alright to use just that there are certain parts where you have to input some information also. I foresee that it might slow down the process a little bit.” (Medical Officer)	“What technology should do, as long as the data is taken in automatically and there's somebody else, I mean, I can't sit and feed all the data. They should employ somebody to put the data in.” (Senior Consultant)

**Social influence** (The degree to which an individual perceives how others believe he or she should use the new system)		

**Professional hierarchy influences decision-making in antibiotic prescribing**	"It makes a difference because if your senior clinician does not want to follow the recommendations, then you as a junior do not have a choice you just follow, because you're not the one making the final decision. You're not the one, the most legally responsible for it." (Medical Officer)	“That's what if you can show us that it's good. Like we have HICC guidelines. We know that this is something developed by our own institution based on our AMR. So, we teach all our registrars and interns please look at this. Or if it's Stanford, we know that this is a quick reference. So, if you can show us a resource, which is useful, there is no reason why we would not use it.” (Consultant)
**Patient perceptions have minimal influence on physicians’ use of an AI-enabled CDSS** **Singapore**: Some physicians felt that some patients would prefer the human touch and the physician's acumen in clinical decision-making, while others felt that patients would be unaware of the use of AI and be unconcerned about the decision-making process. **India**: Patients do not participate in decision-making for antibiotics. Hence, most would not discuss the use of AI with their patients.	“I think most would be encouraged by it. Because technically, it's evidence based, and if it's accurate, it's better than human error … but there may be a percentage will be much smaller, [of] people that will be like, “Oh, you're just trusting in formulations. You're not even diagnosing [as] a doctor.” (Medical Officer) “I think a lot of times patients are not very aware of how we make our decision in terms of antibiotics. They're not fully aware of it at least … I think if we did use this system, I don't think most doctors would say “oh, we use the computer system to decide what kind of antibiotics we should give you.”.” (Medical Officer)	“Why should the patient know we're using AI? So, we are looking at so much of resource. No, even drugs, there are resources. What do you call it ... Drug Information. I don't know, we don't tell the patient that we're using it.” (Consultant)

**Facilitating conditions** **(**The degree to which an individual believes that an organizational and technical infrastructure exists to support the use of the system**)**		

**Workflow integration** Many physicians prefer to integrate the AI-enabled CDSS into their current clinical system for ease of use.	“I think it should be integrated. It's [to do with] ease of drawing the [AI-enabled CDSS] out, and then I presume it would be easier on the back end for the software to draw on all the clinical information of the patient.” (Medical Officer)	“See, Emergency Department, we don't have time. Okay, so if you're going to have multiple things to enter into that app, nobody's going to use it. So, the shorter and the sweeter it is, the better… just don't make it cumbersome. For too many things to fill…I won't just fill in. Because there's too much work and not enough time to fill a lot of facts into a system.” (Consultant)
**Validated and up-to-date algorithms** Most participants expressed the need for rigorous scientific testing and experimentation before such a tool could be incorporated into practice.	“I think they have to try it themselves to see whether it actually works. A lot of times when these things are pushed out and you've never tried it before, on paper it looks good but actually in practice it is very challenging.” (Senior Resident) “So, if there were a central repository of that information that allowed the AI programme to be, updated on a daily basis, that might be cool. I'd have to see if it's able to be validated.” (Senior Consultant)	“But it has to be more categorically proven. That I'm just wondering whether there are RCTs already there or if there are not, why not plan a randomized control trial, one arm with AI, one arm usual care. And you we need to prove that AI is better, in what all ways? Only then we can say it's worth going that way.” (Senior Consultant) “So, I think it has to be field tested here. If we find that it is something I mean like what it's suggesting is what is the best evidence based and cost-effective, then why not?” (India, Consultant)
**Good IT infrastructure** Physicians feel that it is necessary for the institution's hardware (e.g., desktop) to support the processing power required for AI-enabled CDSS.	“I think a good AI will need to have a good set of hardware that can allow this all these calculations to be done without lagging up the whole system. No matter how good or well-intentioned it is, if it takes forever just to load, and the hardware cannot keep up with even the basic processing of the app, people actually do find it burdensome to use.” (Resident)	
**Training and technical support** Support and training prior to implementation.	“Educate. This is assuming they fully trust the system-that it works, and it's accurate. Then it's to educate everyone else that it is accurate. And to show us how it is accurate. So that we are not blindly following." (Medical Officer)	“We will have to have sessions on how to use your app or whatever it is that you're going to provide us. Short of that, where to put the right information inside that app.” (Consultant)
**Voluntary use vs. Mandatory use** Mandating the system will cause resistance in uptake, over-reliance on the system, workflow impediment, and might take away the physician's autonomy.	"I guess making it mandatory would be good but I would see some resistance. I suppose you need to kind of look at readiness from the perspective of the stakeholders, before we decide about making it mandatory." (Consultant) “I think it would interfere if it was made mandatory. ARUS-C already a interferes a little bit, because it is more in the speed of working, and sometimes you've already made a certain decision as to how you want to treat the patient and, [the system] kind of goes against. This new system, I guess it could potentially interfere. The better [the] programme is the less likely it is to interfere.” (Medical Officer)	“Yes, I mean, so, if you make something compulsory, then there is more you know, what do you say, rigidity using it, because you feel you will lose your freedom. But if you say you use it for complex for these types of infections, and people will be more open, I feel.” (Senior Consultant) “We should do it on a trial basis system probably for three to six months and see how it goes. If it was excellent system then all the stakeholders are happy with it, we can even make it to certain extent mandatory and certain extent flexible and open for discussion.” (Consultant)
**Co-creation is necessary** **Singapore**: Co-creation of the AI-enabled CDSS with a sample of representative physicians is lauded by physicians. **India:** Co-creation was mentioned by a few physicians but unimportant.	"Co-creation will allow you to get more buy-in, I feel, right from the start? And then once you get that buy-in then it's easier to push it out to people." (Consultant) "Your sample size needs to be very accurate, so you cannot just be asking senior clinicians, the reason is because senior clinicians may be a bit out of touch in terms of how things work on call." (Medical Officer)	“I think we will have a team meeting and probably in the department discussion and we decide it. I don't think, I don't feel it will be right for some practitioners or even some to develop this, just probably means that there's something about the system that is not reliable.” (Consultant)
**Feedback is necessary and should be prompt** Feedback has to be prompt and taken seriously.	"I think that feedback is important because, if it can be acted on quickly then they are obviously good and tangible outcomes to that are felt on the ground. It's both the issue of work efficiency and also of morale as well." (Resident)	
**Customisability is unnecessary**	"I would say customizability is probably secondary in my mind." (Senior Resident)	
**AI should be updated regularly** Senior physicians stated that the AI-enabled CDSS should be as updated as possible.	"If there were a central repository of that information that allowed the AI programme to be updated on a daily basis, that might be cool. I'd have to see if it's able to be validated." (Senior Consultant)	
**Integration of nationwide data in the AI system** Physicians would like the AI-enabled CDSS to be calibrated with nationwide data.	"What I'm trying to say is that if a patient hospital hops, does it obtain all the culture results from all the other hospitals also? I think it will make a difference because sometimes where the patient might have been- all this would be missed out." (Medical Officer)	
**Easy presentation of AI's rationale** Most physicians expressed that they do not need to understand the inner workings of the AI system but would like the AI-enabled CDSS to provide a short explanation to explain the rationale behind the recommendation.	"I have no understanding of that model." (Medical Officer) "I guess the way of presenting it, I mean to people on the ground, especially physicians are that we don't really need a lot of information like a whole course, but we just need perhaps just a few lines just to explain the rationale." (Resident)	
**Cost-effectiveness considerations** Physicians brought up the need to consider patients' financials.		“It maybe, okay in a simple thing, but patients are never simple. Their financials are never simple. So, if you have multiple options, let's say Amikacin, Piperacillin Tazobactam, so Amikacin, we have much cheaper option for me to give my patient than some. So yeah, multiple options.” Consultant)

**Ethics** (Any other medico-legal or ethical considerations on the use of AI)		

**Prioritizing patient safety** Physicians would prioritise patients' safety over AMR**.**	“For some reason, the AI doesn't take into account that patient had seizure before, and then you follow Ertapenem, and then patient had seizure and patient die from seizure, which we know may or may not be related to Ertapenem lowering the seizure threshold, then whether the AI is responsible or not.” (Senior Resident) "I would be a later adopter rather than an early adopter for the purposes of ensuring that some of these cases are ironed out before I adopt it." (Senior Resident)	“I will take it with a pinch of salt. I'll always take it with a pinch of salt because I trust myself better than a machine, I will definitely consider but I just don't want to totally depend up on.” (Consultant)
**Transparency of AI** Interpretability promotes trust in the AI-enabled CDSS.	“Of course, I mean for everything, [knowing] how you arrive at a certain recommendation- is better. [The recommendation] will be more trustworthy if you know how the system generates all those recommendations.” (Principal Resident Physician)	“Like even choosing an antibiotic you need to take care of the patients…like financial resources, conditions. Many decisions not simple, like some antibiotic resistance and all the bugs, it's not only that you want to make a decision here. There's a lot more things need to go into it.” (Senior Resident)
**Accountability in AI** Some senior physicians mentioned the need for proper and continued oversight of AI-enabled CDSS.	“I think there needs to be a panel of people. I don't know how frequently they should meet up but there should be a determined frequency to evaluate the rules in that AI-derived CDS. Evidence is turning up quite fast sometimes. [As] evidence appears then how [quickly] you meet up and discuss, “Should we build this in, should we not build this in yet?”.” (Consultant)	“And lastly, we need some kind of audit mechanism in place. I'm not sure. Yeah, I guess it should be intradepartmental, which goes upwards to ID or whichever…multiple departments.” (Consultant)
**Supportive medico-legal framework** While most physicians felt that physicians are responsible for medical-legal liabilities as they are the ultimate decision makers, others felt that the hospital should also take responsibility for the AI-enabled CDSS recommendations if the adoption of it is mandatory.	“I guess at the end of the day, if you don't make it a hard stop, the responsibility is in the clinicians because we can choose to follow, or we can choose to override.” (Senior Resident) “I think if you make it mandatory, it is the hospital's responsibility. … If you make it mandatory the risk is that the hospital, then becomes liable." (Senior Consultant)	“I still think the liability is going to be on the physician. I don't think you can pin it on the AI. At least that's what the current law is. Whatever system is used, the liability is physicians.” (Consultant)
**Retaining physicians’ autonomy** Physicians felt that AI should not and will not affect the physicians' autonomy if it is voluntary.	“If you have a system that is built well and can minimize those errors, I'm all for it. Overall, I don't think we should see it as an impediment to the professional autonomy of the clinicians as long as the system is well-built, and it is accurate. And there are no glitches or problems with it. I don't see an issue with it. In fact, I feel it's our duty to embrace such things which would improve patient outcomes.” (Consultant)	“There's some science to it [AI-based tool]. There's some evidence to do it. But I don't think it will completely ever replace the art of going and standing by the bedside and evaluating and changing decisions…I don't think you can ever come to a point when you say the AI will prescribe and doctors can't. If it comes to that, then yes, it's a major infringement of autonomy. And I think all of us will be up in arms.” (Consultant)

*NOTE.* All the themes derived from the interviews are classified under the constructs of the UTAUT model – ‘Performance expectancy’, ‘Effort expectancy’, ‘Social influence’, and ‘Facilitating conditions’. Physicians’ opinions are summarized for each theme and their representative quotes are listed in the columns beside the themes.

### 3.1 Performance expectancy

Most physicians in Singapore felt that the AI tool could facilitate antibiotic prescribing, while most Indian physicians were sceptical about the AI tool's utility. Physicians from both sites were similarly concerned about the impediment of clinical judgement and work efficiency with the use of AI-enabled CDSSs.

#### 3.1.1 Perceived usefulness of AI-enabled CDSS

Most CMCV physicians were sceptical of the utility of an AI-enabled CDSS. They felt that the tool would not value-add to their antibiotic prescribing decisions, given the availability of expert specialists that physicians could consult for difficult decisions. They felt that decision-making for antibiotics is complex and involves considerations which the AI-enabled CDSS may neglect, such as the complexity of multiple morbidities, varied geographies, different susceptibility patterns, and changes in clinical condition. Physicians in CMCV also highlighted patients' economic status as an important consideration for antibiotic decisions and would like the AI-enabled CDSS to consider the patient's socioeconomic status before making antibiotic recommendations.

*“Short answer, it sounds good. But at a practical level, I don't know to what extent that can be useful. I'll tell you the reason because, like for instance, even in our setup, one thing is the huge burden of comorbidities, which most of our patients will have.” (India, Consultant)*

On the contrary, NCID physicians felt that a trusted AI-enabled CDSS would help them arrive at decisions quicker with summarized patient information. The AI-enabled CDSS could also help to affirm physicians’ antibiotic prescribing decisions in some instances.

*“Oh well if it's fast enough, it will definitely be a lot quicker, because now we have to consider all those different things by ourselves right without a system in place but if the system can do it for you and it already takes all that into consideration, it will be much easier.” (Singapore, Medical Officer)*

#### 3.1.2 AI-enabled CDSS may impair clinical judgement

Physicians from both settings were worried that an AI-enabled CDSS might encourage over-reliance on the system and diminish their ability to make prescribing decisions. As NCID and CMCV are teaching hospitals, many senior physicians were concerned that they would be unable to teach younger professionals how to think critically and make decisions on antibiotic prescribing.

*“The thing is, I think that it's not that good for our trainees, because they are not able to make those decisions without the aid of that machine.” (India, Senior Consultant)*

#### 3.1.3 AI-enabled CDSS may impede work efficiency

Some physicians in both sites were concerned that the incorporation of an AI-enabled CDSS in their clinical workflow would slow down processes instead of speeding them up.

*“I may need to type in the information and wait for the system to run. You'll get a bit irritated, because now you need even more input, and most of such black box [systems] they need a bit more computational power so it might be even slower as opposed to a rule-based ARUS-C. So, my main worry is that it slows down the workflow even more.” (Singapore, Medical Officer)*

### 3.2 Effort expectancy

Most NCID physicians felt that minimal effort would be required to learn to use a new AI-enabled CDSS due to familiarity with the rules-based ARUS-C implemented in NCID. However, some NCID physicians and most CMCV physicians were concerned about the additional effort needed to input the required data. They expressed familiarity with the application of AI but could not envisage the integration of an AI-enabled CDSS into their everyday work. Hence, they felt that the tool would be effortful to use.

*“That's what technology should do, as long as the data is taken in automatically and there's somebody else, I mean, I can't sit and feed all the data. They should employ somebody to put the data in.” (India, Senior Consultant)*

### 3.3 Social influence

Social influence comprises physicians’ work relationships and their relationships with their patients. Work relationships influence the adoption of the AI-enabled CDSS in both India and Singapore, but physicians’ impressions of patients’ perceptions differed between both settings.

#### 3.3.1 Professional hierarchy influences decision-making in antibiotic prescribing

Work relationships exert varied influences across different cultures and settings. A few physicians at NCID and CMCV mentioned that they would be influenced by colleagues to use an AI-enabled CDSS at work, with professional hierarchy exerting more influence than peers. Senior physicians and professors at CMCV would encourage their juniors to use the tool if they were using it.

*“It makes a difference because if your senior clinician does not want to follow the recommendations, then you as a junior do not have a choice you just follow because you're not the one making the final decision. You're not the one, the most legally responsible for it so to speak.” (Singapore, Medical Officer)*

#### 3.3.2 Patient perceptions have minimal influence on physicians’ use of an AI-enabled CDSS

Patients do not participate in decision-making for antibiotics in the Indian setting. A patient may discuss the financial implication of using an antibiotic with the doctor but prefers the doctor to make treatment decisions on his/her behalf. Therefore, most Indian physicians did not feel the need to discuss use of an AI-enabled CDSS with their patients.

*“Why should the patient know we're using AI? So, we are looking at so many resources. No, even drugs, there are resources, what do you call them. Drug Information. I don't know, we don't tell the patient that we're using it.”* (India, Consultant)

Shared decision-making is becoming popular in Singapore, although it is still uncommon in antibiotic prescribing in inpatient settings. Some Singaporean physicians felt that patients would prefer the human touch and the physician's acumen in clinical decision-making over an AI-enabled CDSS. Others felt that patients, being unaware of the use of AI in their treatment decisions, would not be overly concerned about the physician's decision-making process. In the case where patients are aware of the doctor's use of an AI-enabled CDSS in their care, one physician felt that most patients would encourage the evidence-based approach, but a small number of them would still prefer the doctor's acumen.

*“I think most would be encouraged by it. Because technically, it's evidence based, and if it's accurate, it's better than human error … but a much smaller percent [of] people will be like, “Oh, you're just trusting in formulations. You're not even diagnosing [as] a doctor.”* (Singapore, Medical Officer)

### 3.4 Facilitating conditions

There were similarities and differences in the conditions that facilitate the adoption of AI-enabled CDSSs in the two institutions.

#### 3.4.1 Workflow integration

Most physicians preferred the AI-enabled CDSS to be integrated with their workflows. They envisioned a well-integrated tool to be one that pulled all relevant information from clinical databases without the need for physicians to input any data.

*“I think it should be integrated. It's [to do with] ease of drawing the [AI-enabled CDSS] out, and then I presume it would be easier on the back end for the software to draw on all the clinical information of the patient.” (Singapore, Medical Officer)*

Physicians in CMCV also expressed that working with a new system is challenging and any change to the existing workflows would be difficult to incorporate if they were on a different platform like the phone.

*“I don't like to begin, to work with any new system, initially maybe a difficult one, but anything if it's got integrated and if the interface is good, we, we will go for it.” (India, Senior Resident)*

#### 3.4.2 Validated and up-to-date algorithms

Most physicians in both sites expressed the need for rigorous scientific testing and validation before an AI-enabled CDSS could be incorporated into clinical practice.

*“So, I think it has to be field tested here. If we find that it is something I mean like what it's suggesting is what is the best evidence based and cost-effective, then why not?” (India, Consultant)*

#### 3.4.3 Training and technical support during implementation and testing

Technical support for troubleshooting and attending to feedback was considered important in the implementation of an AI-enabled CDSS. Physicians in CMCV also mentioned education and increased exposure across departments as important steps in the initial deployment of a new technology.

*“We will have to have sessions on how to use your app or whatever it is that you're going to provide us. Short of that, where to put the right information inside that app.” (India, Consultant)*

#### 3.4.4 Other facilitating conditions that are important to NCID physicians

Other facilitating conditions mentioned by NCID physicians include taking feedback seriously and acting on it promptly, having good IT infrastructure to support the processing power required for the AI-enabled CDSS, and being able to interpret the system's output. Although understanding how the AI system arrived at the recommendations is important, most physicians expressed that they do not need to understand the inner workings of the system but would instead prefer short explanations on the rationale behind the recommendations for them to trust the system.

*"I guess the way of presenting it, I mean to people on the ground, especially physicians is that we don't really need a lot of information like a whole course but we just need perhaps just a few lines just to explain the rationale." (Singapore, Resident)*

Co-creation of the AI-enabled CDSS with a sample of representative physicians is lauded by NCID physicians. However, they did not find customizability necessary. Co-creation was also welcomed by a few physicians in CMCV.

*“So, co-creation will allow you to get more buy-in, I feel, right from the start? And then once you get that buy-in then it's easier to push it out to people.” (Singapore, Consultant)*

#### 3.4.5 Adoption of AI-enabled CDSSs to be voluntary instead of mandatory

Mandating the use of AI tools may cause resistance in uptake, over-reliance on the system, workflow impediment, and loss of the physician's autonomy. Physicians in CMCV also expressed that they were more likely to adopt the AI-enabled CDSS if they had the freedom to explore the tool and discover its benefits than if they were mandated to use it.

*“I guess, making it mandatory would be good but I would see some resistance. I suppose you need to kind of look at readiness from the perspective of the stakeholders before we decide about making it mandatory." (Singapore, Consultant)*

*“We should do it on a trial basis system probably for three to six months and see how it goes. If it was excellent system then all the stakeholders are happy with it, we can even make it to certain extent mandatory and certain extent flexible and open for discussion.” (India, Consultant)*

### 3.5 Ethical considerations

Ethical considerations are not part of the UTAUT model but are essential for the use of AI in medicine for patients’ safety and to facilitate its acceptance and adoption. Prioritizing patient safety, transparency and accountability of AI, retaining physicians’ autonomy, and having a supportive medico-legal framework are themes that emerged from the interviews.

#### 3.5.1 Prioritizing patient safety

An AI-enabled CDSS tool would need to perform adequate risk assessments before making antibiotic recommendations, to ensure patient safety and physician confidence in its use. Some physicians felt that the considerations of the AI-enabled CDSS could be limited and may compromise patient safety.

*“For some reason, the AI doesn't take into account that patient had seizure before, and then you follow Ertapenem, and then patient had seizure and patient die from seizure, which we know may or may not be related to Ertapenem lowering the seizure threshold, then whether the AI is responsible or not.” (Singapore, Senior Resident)*

#### 3.5.2 Transparency and accountability of AI

Transparency in AI is fundamental to its interpretability, which in turn promotes trustworthiness in the AI system. Some physicians from both sites would like to have information on how the recommendations were made by the AI-enabled CDSS to trust its recommendations.

*“Of course, I mean for everything, [knowing] how you arrive at a certain recommendation- is better. [The recommendation] will be more trustworthy if you know how the system generates all those recommendations.” (Singapore, Principal Resident Physician)*

Senior physicians in both sites also mentioned the need for proper and continued oversight of AI-enabled CDSSs for accountability. Audits and evidence reviews are important to ensure that the recommendations made by the AI-enabled CDSSs are relevant and up to date.

*“We need some kind of audit mechanism in place. I'm not sure. Yeah, I guess it should be intradepartmental, which goes upwards to ID or whichever…multiple departments.”* (India, Consultant)

#### 3.5.3 Supportive medico-legal framework

A supportive medico-legal framework is essential to instill confidence in AI adoption in clinical decision-making, as liabilities associated with AI-enabled technologies are not yet established or well regulated. While most physicians felt that they are responsible for medico-legal liabilities as the ultimate decision-makers, others felt that the hospital should take responsibility for recommendations made by AI-enabled CDSSs, if their adoption were made mandatory.

*“I think if you make it mandatory, it is the hospital's responsibility. … If you make it mandatory the risk is that the hospital then becomes liable.”* (Singapore, Senior Consultant)

*“I still think the liability is going to be on the physician. I don't think you can pin it on the AI. At least that's what the current law is. Whatever system is used, the liability is physicians.”* (India, Consultant)

#### 3.5.4 Retaining physicians’ autonomy

Most physicians in NCID were not concerned about AI infringing physicians’ autonomy as they are the final decision makers regarding their patients’ care. They felt that an AI-enabled CDSS would not affect physicians’ autonomy if its adoption is voluntary. Physicians were more concerned about the accuracy of the AI-enabled CDSS than infringement of physicians’ autonomy.

*“If you have a system that is built well and can minimize those errors, I'm all for it. Overall, I don't think we should see it as an impediment to the professional autonomy of the clinicians as long as the system is well-built, and it is accurate. And there are no glitches or problems with it. I don't see an issue with it. In fact, I feel it's our duty to embrace such things which would improve patient outcomes.” (Singapore, Consultant)*

Physicians in CMCV were more sceptical about the AI-enabled CDSS making complex decisions for antibiotic prescribing. They felt strongly that the CDSS should not replace a physician's decision-making process and autonomy.

*“There's some science to it [AI based tool]. There's some evidence to do it. But I don't think it will completely ever replace the art of going and standing by the bedside and evaluating and changing decisions. I don't think you can ever come to a point when you say the AI will prescribe and doctors can't. If it comes to that, then yes, it's a major infringement of autonomy. And I think all of us will be up in arms.” (India, Consultant)*

## 4 Discussion

This study explored the perceptions and concerns of physicians in India and Singapore on an AI-enabled CDSS for antibiotic prescribing and identified the barriers and facilitators for implementation. Physicians in Singapore were more accepting of such a tool, while physicians in India were sceptical about the value of an AI-enabled CDSS in helping them to make antibiotic prescribing decisions. Notable facilitating conditions at both sites include integrating the CDSS with the physicians’ workflows, validating the accuracy of the CDSS's algorithms and antibiotic recommendations, and training in and technical support for the use of the system. Other essential factors influencing the adoption of an AI-enabled CDSS include co-creating the tool with physicians and allowing voluntary use**.** To our knowledge, this study is the first physician-focused qualitative study that has explored the acceptance of AI-enabled CDSSs in different cultural and practice contexts. By comparing the perceptions of physicians in India and Singapore, we gained deeper insights and a better understanding of the concerns and factors influencing the acceptance of such tools that can be generalized to other countries.

Familiarity with digital technology in healthcare influences physicians' acceptance of integrating a new technology in daily clinical practice. Global comparisons of digital adoption demonstrate a digital divide between high income countries and LMICs [27], and India and Singapore are observed to be at different ends along the spectrum of digital health technology adoption. In our study, we noted that the optimism among NCID physicians about the utility of AI-enabled CDSS stems from the experience of using a rules-based CDSS (ARUS-C) in current day-to-day clinical practice. Solutions targeted at digital inclusion and equity, tailored to the local context, could potentially narrow the gap in digital divide.

Our study illustrated the importance of study context in shaping the acceptability of the AI-enabled CDSS. With regard to desirable features of AI-enabled CDSSs, a unique theme from the interviews with Indian physicians was the tool's ability to provide an antibiotic of choice after weighing the cost of medicines with the socioeconomic situation of the patient. A recent study about physicians’ perceptions on the use of an AI-enabled CDSS for oncology across India, China, Brazil, South Korea, and Mexico also reported cost consideration as a desirable feature [28]. This theme relates to the local context of poverty and out-of-pocket expenditure for medicines in India which is vastly different from the context in Singapore. The difference in physicians’ acceptance of an AI-enabled CDSS for antibiotic prescribing in India and Singapore further highlights the relevance of studying the context in which innovations will be implemented.

Conditions that facilitate physicians’ intention to adopt an AI-enabled CDSS centered around factors that enhance users’ experience (i.e., workflow integration, IT infrastructure, ease of use, adequate training and support). These factors are essential for successful implementation but might be constrained by the availability of resources. Regardless, the AI tool needs to be tested and validated to instill confidence in physicians to increase its uptake [29]. The loss of autonomy in clinical decision making and the concern that the AI tool could impede a physician's clinical judgement was a common theme in both India and Singapore. Apart from the fear of losing autonomy in healthcare, it is often cited as the biggest threat to future developments in AI tools for everyday life [30]. However, a complete reliance on AI is unlikely to happen because physicians are liable for their clinical decisions. On the contrary, AI could help physicians address administrative issues, overcome decision fatigue, and solve complex problems. Nonetheless, physicians’ concerns about these issues may implicate AI implementation in healthcare and should be addressed early in the implementation cycle.

Work relationships exert limited influence on physicians’ intention to adopt an AI-enabled CDSS in our study. Although most participants indicated that their decision to use an AI tool is independent of work relationships, seniors in the medical profession may influence juniors to adopt AI if they use it. Hence, getting buy-in from senior physicians is crucial for the implementation and scaling of AI-enabled CDSSs.

Many participants felt the non-necessity to discuss AI-based decision support with their patients as most patients would be oblivious to the decision-making mechanisms physicians use for their treatment. In the Asian context, doctors assume a paternalistic role in decision-making due to patients’ prioritisation of the value of health over autonomy [31,32]. Most patients in India still prefer physicians to make treatment decisions on their behalf [32], but in Singapore, there is a gradual shift from paternalism to more shared decision-making between patients and physicians as health literacy increases in the population [31]. Given the fiduciary nature of the patient-physician relationship and the spectrum of decision-making preferences, ethical frameworks are necessary to safeguard patient interests [33].

Technology adoption often lags in health care because of patient safety concerns, regulatory barriers, and other ethical considerations. Although the UTAUT model has been widely used to explain user intention to adopt technology, the model failed to account for medico-legal and ethical considerations that may arise from AI use. These considerations are some reasons for hesitancy in AI adoption among our participants. Therefore, medical boards and institutions need to step up the oversight of AI to enable its implementation. While many countries have developed frameworks to guide the ethical use of AI, the European Union is the only entity with the AI legal act [34]. Singapore has recently launched the A.I. Verify—the world's first AI governance testing framework and toolkit for companies to demonstrate and verify responsible AI [35]. This move is a step forward to instill confidence in the providers and consumers of AI.

This study provided insights relevant to implementing AI-enabled decision-support tools in health care. However, the study is limited by providing perspectives only from physicians. Patients’ and administrators’ perspectives would have added to a more holistic understanding of the use of AI in health care settings. Another study limitation is the coverage of only one centre in India and one centre in Singapore, and the differences in the specialties of the centres in both countries. Sampling to include more than one institution in each country would have added greater breadth and variation to the sample. Because the progression and widespread use of AI is inevitable, future studies should consider assessing the ethical and medico-legal concerns of AI-enabled CDSS and include physicians with more diverse technological backgrounds.

## 5 Conclusions

In conclusion, the acceptance of AI-enabled CDSSs depends on the physician's confidence with the tool's recommendations, perceived ease of use, familiarity with AI, the organisation's digital culture and support, and the presence of medico-legal governance of AI. New AI-enabled technologies should be tailored to the local practice context in health care to facilitate their acceptance and adoption. Progressive implementation of evidence-based best practices and continuous feedback are essential to allay scepticism around the utility of AI-enabled CDSSs.

## Appendix Supplementary materials

Image, application 1
