# Supplementary material for: Are physicians ready for precision antibiotic prescribing? A qualitative analysis of the acceptance of artificial intelligence-enabled clinical decision support systems in India and Singapore
Source: J Glob Antimicrob Resist. 2023 Dec;35:76–85. doi: 10.1016/j.jgar.2023.08.016 (PMC10684720; doi:10.1016/j.jgar.2023.08.016)
Supplement: Supplementary file 1 [file mmc1.docx]

**Supplementary materials**

**S1a: India’s study context**

India, the world's largest democracy with a population above 1.2 billion, has made enormous contributions to medicine especially during the COVID-19 pandemic. Like other LMICs, India is characterised by a society in demographic and epidemiologic transition. While there is a rapid transition in disease patterns from communicable diseases to chronic non-communicable diseases (NCDs), the burden of communicable diseases remains high[1]. The dual burden of disease has overburdened the health system and its limited resources. AMR has emerged as a serious challenge due to the limited laboratory capacity for aetiology based targeted treatments and the wide availability of relatively inexpensive antibiotics.

Recent estimates from the Indian council of medical research (ICMR)’s AMR surveillance confirm an increase in resistance patterns in 2019 [2]. Although the ICMR has a technologically robust surveillance system in place and has initiated programs and standardised guidelines to tackle the problem of AMR, the irrational use of antibiotics persists in the population [3]. While guidelines are available, there are no decision support (CDSS) tool that are widely used to assist rational, evidence based antibiotic decision making. AI-based decision support tools may be a promising solution for rational antibiotic prescribing. The current use of AI in clinical decision-making is limited, with the main barrier being the lack of adequate infrastructure, concerns about data security and confidentiality in AI-based tools. Further, much of the cloud-computing infrastructure and servers required are located outside India.

**S1b: Singapore’s study context**

Singapore, a densely populated island nation with a population of 5.7 million, has achieved remarkable improvement in health outcomes despite a relatively low healthcare expenditure (<5% of gross domestic product) compared with other high-income countries [4].

Under the National AI Strategy, many AI projects have been implemented in healthcare. For example, two of three public healthcare clusters in Singapore have transited their electronic medical records (EMR) to the EPIC system to leverage the benefits of AI and reduce data fragmentation [5]. Given the increased use of AI in healthcare, the Ministry of Health has co-developed guidelines (e.g., “AIHGIe”) with several government-backed institutions in Singapore to govern patient safety and increase trust in the use of AI in healthcare [6].

AI has not been implemented in hospital routine care to tackle AMR in Singapore, although ASPs were implemented in the public hospitals since 2011 with favorable outcomes on length of hospital stay and antibiotic use[7]. Tan Tock Seng Hospital (TTSH) was among the first few hospitals in Singapore that implemented a rules-based CDSS —Antimicrobial Resistance Utilization and Surveillance Control (ARUS-C) interface—to guide antibiotic selection and dosing [8]. The hospital’s antimicrobial stewardship committee considered the local epidemiology of infectious diseases, local microbiology, and antibiotic susceptibility patterns in the hospital in the past five years, and international guidelines when developing ARUS-C. Physicians can seek antibiotic prescribing guidance from ARUS-C, which takes into account the patient’s antibiotic allergies and renal function and recommends the narrowest-spectrum antibiotic for common organisms responsible for the diagnosed infection. The system automatically launches when physicians make an electronic broad-spectrum antibiotic prescription that is under stewardship [8]. However, the rules-based ARUS-C might not address the antibiotic needs of patients with complex clinical conditions. Therefore, an AI-based CDSS is desired to address the current limitations of ARUS-C [8].

**S2: Singapore National Centre for Infectious Diseases (NCID) — interview vignette**

The NCID team presented a proof of concept of an AI-enabled Clinical Decision Support System (CDSS) by comparing it with an existing rules-based CDSS to guide physicians in antibiotic prescribing in NCID. The existing rules-based CDSS is known as the Antimicrobial Resistance Utilisation and Surveillance Control (ARUS-C) system, introduced to guide antibiotic selection and dosing. We used a vignette to illustrate our case. **Figures 1a – 1d** show the clinical case study and mock-up interface of the team’s proof of concept of an AI-enabled CDSS.

- A 72-year old woman with a urinary catheter in situ presents to the emergency department with fever (38.5) and right flank pain.
- Her urine dipstick was positive for leucocytes and nitrites, and her working diagnosis septic shock with acute kidney injury.
- Urine and blood cultures were ordered and the physician would like to start patient on empiric antibiotics.
- Currently, her serum creatinine is 148 µmol/l and her weight is 53 kg.
- The E.coli bacteria noticed in the urine cultures 1 month ago was resistant to oral ciprofloxacin, amoxicillin and cefazolin.

.

**Figure 1a:** Clinical case study


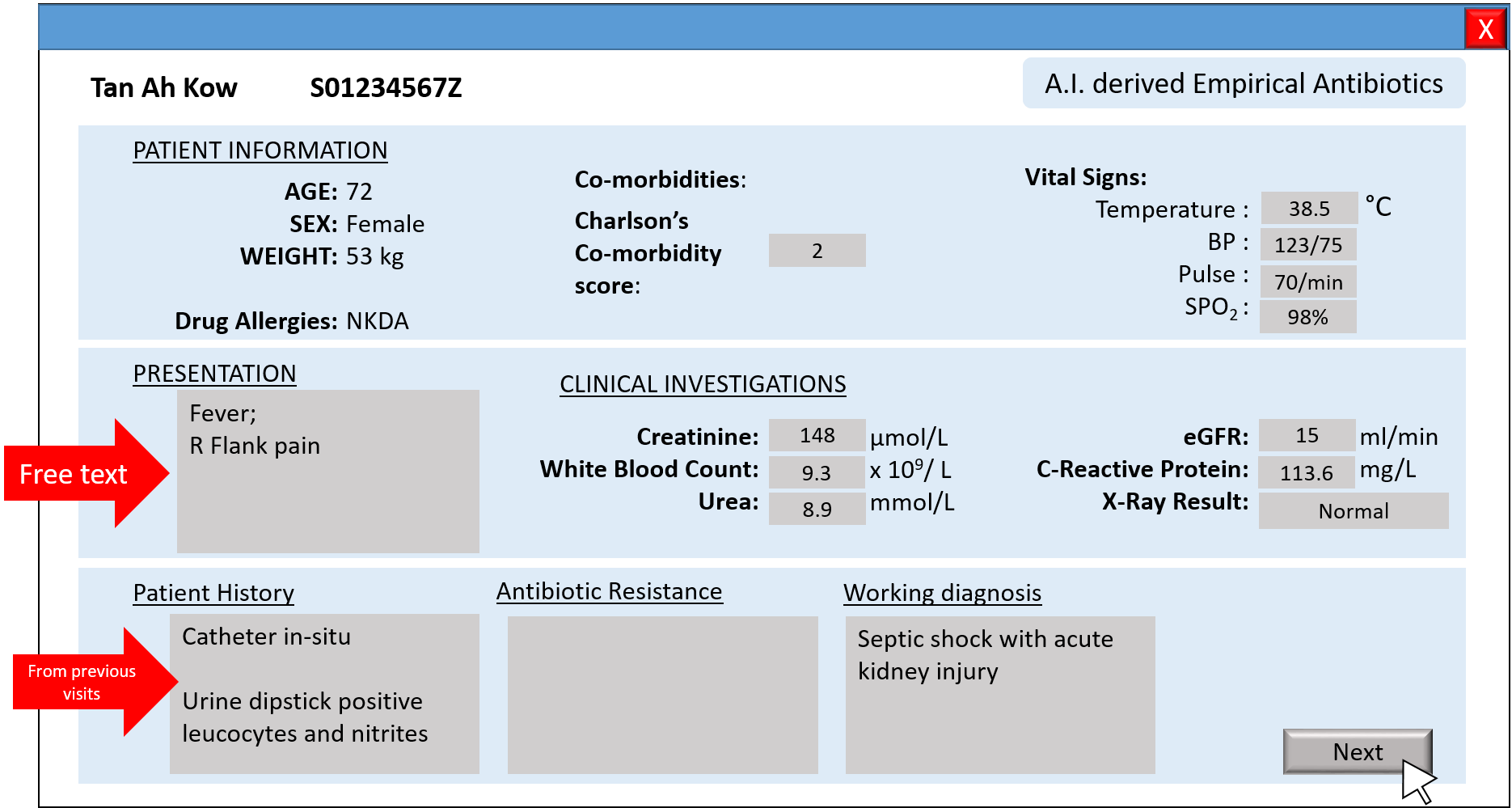


**Figure 1b:** Mock-up interface of patient details and clinical presentation based on the clinical case study presented in Figure 1a


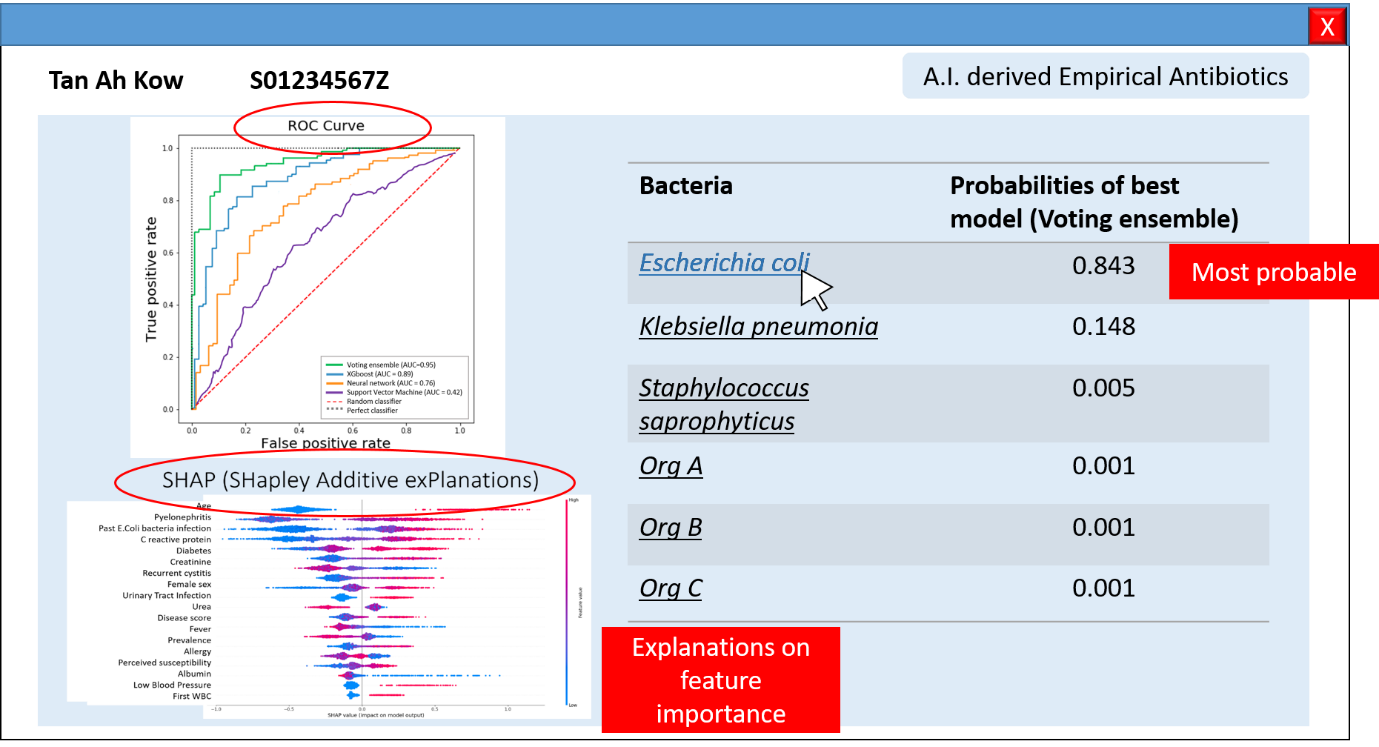


**Figure 1c:** Mock-up interface of our AI-enabled CDSS displaying an output of the probable organisms causing the patient’s infections. The interface shows the ROC curves and a SHAP plot to explain the features used to predict the model.


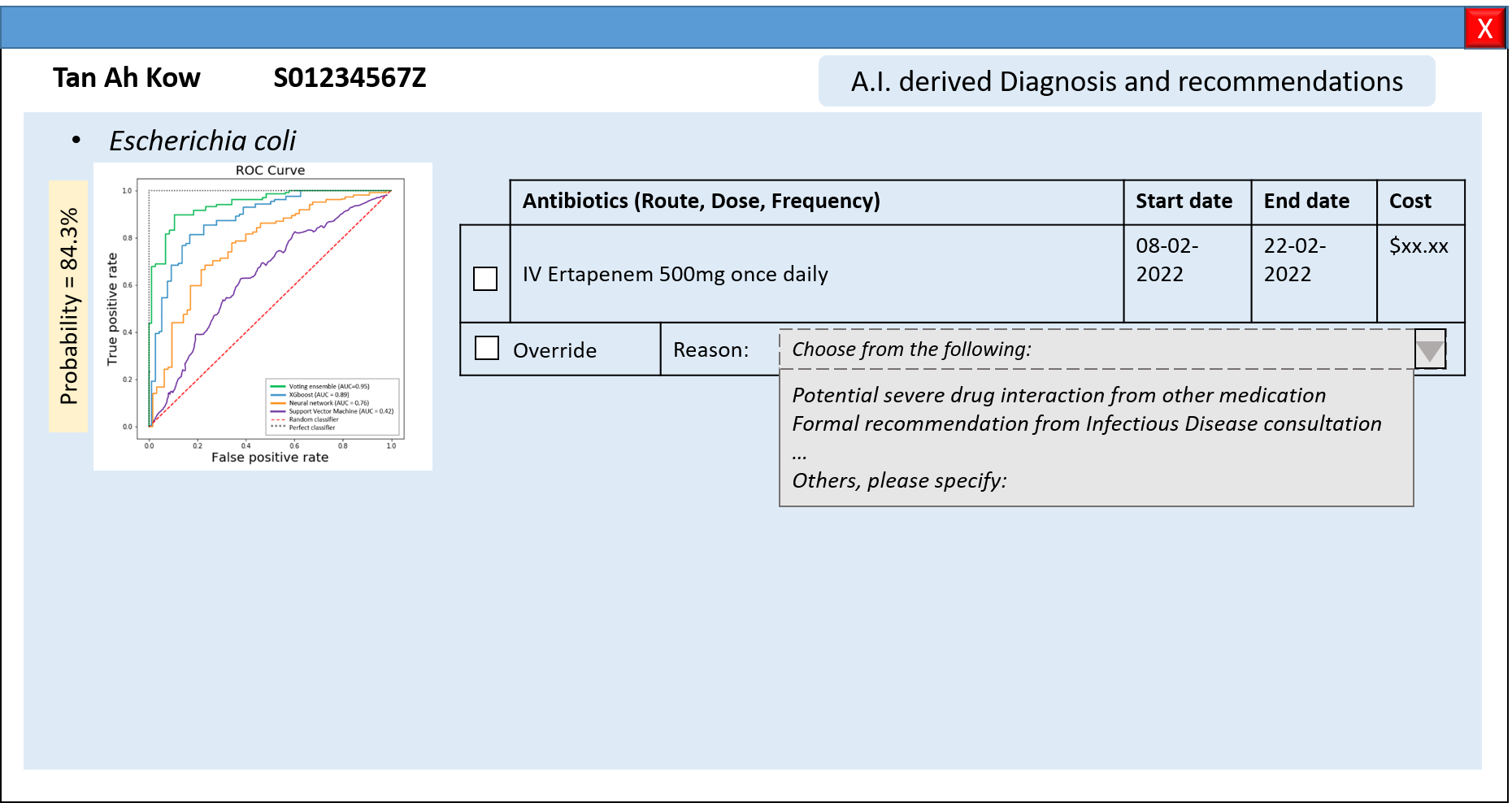


**Figure 1d:** Mock-up interface of our AI-enabled CDSS displaying the proposed empirical antibiotic for the clinical case study in Figure 1a.

**S3: India Christian Medical College Vellore (CMCV) — interview vignette**

The Vellore team presented the workings of and potential benefit of and AI-enabled CDSS to their participants before presenting a clinical case vignette. Figure 2 shows the vignette used for discussion. Not all participants were presented the vignette in these interviews.

- A 28-year-old lady presented with complaints of high-grade fever, dysuria and right flank pain for 2 days during 28 weeks of pregnancy. So far, her pregnancy is uncomplicated by any medical problems.
- You are seeing her in the emergency department.
- Clinical examination revealed tachycardia, tachypnoea and severe right renal angle tenderness. Her bloods are as follows: WBC: 14,100/cu mm (N 81, L 17, Band forms 2)
- Creatinine 2.1mg/dl, Urine micro – numerous WBCs
- Ultrasound abdomen: bulky kidney with mild increase in cortical echogenicity.
- There is no obstruction/perinephric collection.
- Blood & Urine cultures were sent.

**Figure 2:** Vignette used by CMCV for discussion.

**S4: Semi-structured interview guide used by NCID and CMCV**

Table 1 shows the interview questions extracted from the NCID and CMCV interview guides.

**Table 1:** Qualitative interview guide

| **Qualitative Interview Guide** |
| --- |
| **Understanding of Artificial Intelligence (AI) in clinical decision support systems (CDSS)** |
| • Can you share with me what you know about Artificial Intelligence (AI) in decision making?  • Can you share with me your experiences of using the ARUS-C CDSS tool? *(NCID only)* |
| • What is your general perception of using AI in healthcare? Do you have any past experiences? *(CMCV only)* |
| **Trust in clinical decision of AI** |
| • Would you be confident/trust the recommendations of an AI-based CDSS? Why/why not?  • Some have mentioned that AI is a black box system that makes it difficult to understand the pathways the system takes to arrive at the output. What are your thoughts on this? |
| **Performance expectancy** |
| • How useful do you think such a system (or tool) could be for you to make decisions on antibiotic treatment (i.e., type, duration, and whether antibiotics are required) for your patients? |
| • How do you think the use of such an AI-enabled system will affect your work productivity? |
| **Effort expectancy** |
| • What do you think of the ease of use of such an AI-based system?  • What are your thoughts on learning to use such a new system in your work? |
| **Social influence** |
| • How inclined would you be to use the system if your peers are using it?  *Probe for the different kinds of relationship, e.g., senior/junior/peers* |
| • How do you think patients will perceive you using such a system in making recommendations for their treatment? |
| **Facilitating conditions** |
| • What do you think is needed from the management/institution to successfully implement such a system?  *Probe for interoperability and type/frequency of training* |
| • How do you see such a system integrating with your existing system?  • Do you think training and tech support is needed for deployment of such a system? Why/why not? |
| **Ethics** |
| •What are your thoughts about AI and clinician’s professional autonomy?  •Assuming the patient deteriorates due to recommendations of the system, would you be concerned about medical-legal liabilities associated with using the system? Why/why not?  •Who do you think should be responsible for any medical-legal issues arising from use of such a system?  •What legal frameworks/regulations do you think need to be in place? |
| **Behavioural intention** |
| • If the CDSS mentioned above was to be implemented in our institution in the next month voluntarily, would you use it?  • What are some reservations you might have in using such a system? How can it be overcome? |
| **Desirable features in AI** |
| • What are some features you look for in such a technology interface?  *Probe around adaptability/customization, feedback loop, co-creation*  • After our discussion, would you prefer to use a rules-based clinical decision support system or an AI-based system? |

**References**

1. India State-Level Disease Burden Initiative Diabetes C. The increasing burden of diabetes and variations among the states of India: the Global Burden of Disease Study 1990-2016. Lancet Glob Health. 2018;6(12):e1352-e62.

2. IMCR. India's Antimicrobial Resistance Surveillance & Research Initiative: Government of India; 2022 [cited 2023. Available from: <https://iamrsn.icmr.org.in/>.

3. Vijay S, Sharma M, Misri J, Shome BR, Veeraraghavan B, Ray P, et al. An integrated surveillance network for antimicrobial resistance, India. Bull World Health Organ. 2021;99(8):562-71.

4. Ramesh M, Bali AS. The Remarkable Healthcare Performance in Singapore. 2019 [cited 12/14/2022]. In: Great Policy Successes [Internet]. Oxford University Press, [cited 12/14/2022]; [0]. Available from: <https://doi.org/10.1093/oso/9780198843719.003.0003>.

5. Tan Tock Seng Hospital. Transforming Care with the Next Generation Electronic Record (NGEMR) System 2022 [15th December 2022]. Available from: <https://www.ttsh.com.sg/About-TTSH/Pages/NGEMR.aspx>.

6. Ministry of Health. ARTIFICIAL INTELLIGENCE IN HEALTHCARE 2022 [14th December 2022]. Available from: <https://www.moh.gov.sg/licensing-and-regulation/artificial-intelligence-in-healthcare>.

7. Loo L, Lee W, Chlebicki P, Kwa AL. Implementing National Antimicrobial Stewardship Program (ASP): Our Singapore Story. Open Forum Infectious Diseases. 2016;3(suppl_1):1678.

8. Chow AL, Lye DC, Arah OA. Patient and physician predictors of patient receipt of therapies recommended by a computerized decision support system when initially prescribed broad-spectrum antibiotics: a cohort study. Journal of the American Medical Informatics Association. 2015;23(e1):e58-e70.
